# Temperature-Driven Responses and Contributions of Hyperthermophiles: Linking Storage and Inoculation Strategies in Municipal Sludge Composting

**DOI:** 10.3390/microorganisms14051064

**Published:** 2026-05-08

**Authors:** Zixi Ming, Shihong Chen, Jun Gu, Ran Yu

**Affiliations:** 1 Department of Environmental Science and Engineering, School of Energy and Environment, Southeast University, No. 2 Southeast University Street, Nanjing 210096, China; mingzixi2023@163.com (Z.M.); waxptds@163.com (S.C.); gujun@seu.edu.cn (J.G.); 2 Key Laboratory of Environmental Medicine Engineering, Ministry of Education, Southeast University, Nanjing 210009, China

**Keywords:** aerobic composting, bioaugmentation, hyperthermophilic consortium, medium-temperature inoculation

## Abstract

Conventional aerobic composting is limited by incomplete organic matter degradation, long composting times, and low product quality. Hyperthermophiles have been applied in composting, but systematic studies on their storage conditions and inoculation strategies are lacking. In this study, the hyperthermophilic microbial consortium, designated as NJ, maintained higher post-storage regrowth capacity after 6-month storage at 25 °C and 4 °C than at −80 °C. Furthermore, inoculated at the medium-temperature stage, NJ enhanced organic matter decomposition and shortened the composting time by 50% compared with high-temperature stage inoculation (>55 °C). Compared with a commercial inoculant, NJ shortened composting time by 67%, increased the germination index from 70% to 85%,raised DTN by 40%, and led to humic substance accumulation by the end of composting, indicating improved product quality. Consequently, medium-temperature stage inoculation of NJ enhances composting efficiency and product quality by enabling earlier functional expression and effective ecological niche occupation.

## 1. Introduction

Municipal dewatered sludge is an important by-product of wastewater treatment that contains beneficial nutrients but also harmful constituents, such as pathogenic microorganisms, organic pollutants, and heavy metals [1]. Aerobic composting is a widely used approach for municipal sludge treatment. However, traditional aerobic composting is often constrained by slow heating rates, low efficiency of organic matter degradation, incomplete pathogen inactivation, and long composting times [2].

In recent years, bioaugmentation with hyperthermophiles has been increasingly applied in sludge composting to enhance performance. Studies have shown that hyperthermophile inoculation can significantly accelerate heating rates and organic matter degradation [3]. For instance, in a large-scale composting system composed of municipal sludge and food waste, hyperthermophiles were applied, resulting in the center temperature of the pile exceeding 100 °C and the isolation of a member of the genus *Calditerricola*, suggesting the ecological functions of these microorganisms during composting [4].

Hyperthermophiles are generally considered advantageous for composting enhancement because they can maintain enzymatic activity and support continuous organic matter degradation under extremely high-temperature conditions (e.g., above 80 °C) [5]. These adaptations are associated with multiple physiological and molecular mechanisms, including membrane stabilization, enhanced protein structural rigidity, and nucleic acid protection, which collectively support microbial activity under extreme temperatures [6,7,8,9]. These diverse thermophilic characteristics not only explain the persistence of hyperthermophiles under extreme conditions but also suggest that inoculation stages may interact with specific mechanisms, thereby resulting in different composting performances. Previous studies have reported that hyperthermophilic consortium can extend the high-temperature period and improve humification [10]. However, despite these advances, limited attention has been paid to how storage conditions influence post-storage regrowth capacity of hyperthermophilic consortium, and how the inoculation stage interacts with community adaptation to shape composting outcomes. In particular, the linkage between storage strategy, microbial community succession, and composting performance remains insufficiently understood.

Therefore, the main objectives of this study are to: (1) screen hyperthermophile consortium (NJ) and assess its post-storage regrowth capacity at different temperature conditions; (2) examine the composting effects of NJ inoculation at different composting stages; (3) compare the composting effects of NJ inoculation with those of a commercial inoculant.

## 2. Materials and Methods

### 2.1. Consortium Sources

The NJ used in this study was screened from a 90–95 °C geothermal hot spring and identified as an aerobic hyperthermophilic microorganism consortium based on its growth characteristics at 80 °C and 16S rRNA gene sequence analysis. The commercial inoculant (SY) was a product purchased from the Henan Haowangnong Biotechnology Co., Ltd., Zhengzhou, China., with a microbial concentration of 5 × 10^8^ CFU/mL. According to the manufacturer’s instructions, SY was applied at 0.1% (*v*/*v*) of the composting substrate. To ensure comparability, the inoculation concentration and proportion of NJ in the experimental group were adjusted to match the total microbial input of the commercial inoculant. Raw sequence data were submitted to the NCBI Sequence Read Archive (accession no. PRJNA1395395).

### 2.2. Pilot-Scale Composting System

The pilot-scale composting platform is shown in the Supplementary Information (Figure S1). The composting bin (2 m × 1 m × 1 m, L × W × H) accommodated approximately 400 kg of materials. Temperature probes and oxygen sensors at the pile center continuously monitored conditions, with data transmitted to a Programmable Logic Controller (PLC). Aeration and turning were controlled via an interlocking logic program. Operational parameters are provided in the Supplementary Information (Table S1).

### 2.3. Experimental Methods

#### 2.3.1. Isolation and Cultivation of Hyperthermophiles

Hot spring samples were pretreated as previously described and inoculated into Cysteine–Yeast–Sucrose (CYS) medium at a 5% (*v*/*v*) ratio [11]. The consortium was cultivated in a water bath at 80 °C, with turbidity monitored daily and cell density recorded microscopically. Microbial cell density was expressed as total cell concentration. Briefly, the culture samples were vortexed for 30 min, allowed to stand for 30 min, and the supernatant was collected for microscopic counting using a hemocytometer. Once the cell density plateaued, 3% (*v*/*v*) of the cultures was transferred into fresh CYS medium, and cultivation was continued under the same conditions. After reaching stable cell density, the temperature was adjusted to above 80 °C to compare cell density across temperatures and determine the optimal growth temperature.

#### 2.3.2. Storage Conditions for Hyperthermophiles

A 0.5 mL aliquot of a logarithmic-phase culture was mixed with 0.5 mL of sterile 30% (*v*/*v*) glycerol, resulting in a final concentration of 15% (*v*/*v*) as a cryoprotectant, and this procedure was applied consistently across all experimental groups [12]. The mixture was transferred into sterile cryotubes and stored at −80 °C, 4 °C, and 25 °C for 6, 9, and 12 months, respectively, to evaluate the storage stability of the cultures. Storage at −80 °C was selected as a conventional method for long-term microbial preservation, while temperatures of 4 °C and 25 °C were selected to represent typical short-term and room-temperature storage conditions, respectively. These conditions were used to evaluate whether the hyperthermophilic consortium exhibits distinct storage behavior compared to conventional microorganisms. At the end of the storage period, the preserved cultures were thawed at 30 °C and inoculated into CYS medium at 5% (*v*/*v*), followed by static incubation at 80 °C for 7 days. The temperature of 80 °C was selected as a standardized recovery condition, representing the lower threshold of hyperthermophilic growth, rather than the optimal growth temperature. In practical applications, preserved microbial cultures are typically reactivated and propagated prior to use. Therefore, the present study aimed to evaluate the post-storage recovery process, rather than direct survival. Cultures were successively subcultured three times at 3% (*v*/*v*) inoculation to allow recovery and stabilization after storage. The maximum cell density was then determined using microscopic counting with a hemocytometer. This approach reflects the post-storage regrowth capacity of the microbial community. The hyperthermophilic nature of NJ was determined based on its ability to resume active growth and metabolic activity upon reinoculation at 80 °C, which is within the optimal temperature range for hyperthermophiles. Viability after storage at 25 °C reflects survival under suboptimal mesophilic conditions rather than active growth and therefore does not alter its classification as a hyperthermophilic consortium.

#### 2.3.3. Experimental Design of Inoculation at Different Stages with NJ

Two experimental groups were established: a medium-temperature stage group (medium-T), with NJ inoculated at the start of the composting process, and a high-temperature stage group (high-T), with NJ inoculated after the temperature exceeded 55 °C. In addition, corresponding ambient medium-T and ambient high-T data were included. The term “ambient temperature” refers to the surrounding environmental air temperature recorded during the experimental period that was not artificially regulated and was used as a reference for comparison with the composting temperature profiles. Sludge and straw were used as composting materials in all treatments. The same raw materials and mixing ratios were applied throughout the experiments. Detailed substrate compositions are provided in the Supplementary Information (Table S2). In Section 2.3.2, 80 °C was selected as the laboratory recovery temperature to verify the ability of NJ to resume active growth under hyperthermophilic conditions. In contrast, in Section 2.3.3, 55 °C was selected as the inoculation temperature because it approximates the upper limit typically reached during conventional composting processes. Since most conventional composting microorganisms cannot survive at temperatures as high as 80 °C, such conditions are rarely achieved in practice. Therefore, inoculation at 55 °C allows the introduced hyperthermophilic consortium to become active under realistic composting conditions and potentially elevate the system beyond the conventional thermophilic range.

Samples were collected during the medium-temperature stage, high-temperature stage, cooling stage, and maturation stage. At each point, three independent composite samples were collected from each composting pile using a five-point method and homogenized. A total of 10 g was stored at −20 °C for microbial analysis, while the remaining fresh samples were used for physicochemical measurements or air-dried and stored at 4 °C for elemental analysis. All parameters were measured in triplicate, and results are presented as mean ± standard deviation.

#### 2.3.4. Composting Effects of NJ Inoculation vs. Commercial Inoculant

To evaluate the NJ performance at the medium-temperature stage (medium-T), both NJ and SY were inoculated at the beginning of composting. SY was employed as a control to enable comparison under identical inoculation conditions. Substrate preparation, compost composition, and experimental parameters were consistent with those described in Section 2.3.3. Detailed compositions are provided in the Supplementary Information (Table S3). All piles were inoculated with microbial agents. The primary aim of this study was to investigate the process optimization during ultra-thermophilic composting using NJ and to compare its performance with that of SY across different composting stages, rather than to quantify absolute effects relative to non-inoculated (natural) compost.

### 2.4. Measurement of Physicochemical Parameters

The compost temperature, oxygen concentration, moisture content, pH, and electrical conductivity (EC) were measured following previously reported methods [13]. Germination index (GI) was determined according to the Chinese national standard NY/T 525-2021 [14] for organic fertilizers. Dissolved organic carbon (DOC) was measured in the leachate using a Total Organic Carbon (TOC) analyzer. Soluble ammonium nitrogen was measured by the Nessler’s reagent colorimetric method, and soluble nitrate nitrogen was determined using UV spectrophotometry [15]. Urease activity was assessed spectrophotometrically at 578 nm [16]. The detailed method for the determination of total humic substances is provided in the Supplementary Information (Text S1).

### 2.5. Microbial Community Identification and Analysis of Compost Sample Community Structure

Bacterial suspensions were obtained by mixing 10 g of the compost sample with 90 mL of sterile PBS and shaking thoroughly. The suspension was then centrifuged at 10,000 rpm for 3 min at room temperature, and the sediment was inverted on blotting paper for 1 min to remove residual liquid. DNA was extracted using the E.Z.N.A™ Mag-Bind Soil DNA Kit (Omega Bio-tek, Norcross, GA, USA), checked for integrity using agarose gel electrophoresis, and the DNA sample concentration was quantified using a Qubit fluorometer (Thermo Fisher Scientific, Waltham, MA, USA). The V4-V5 region of the bacterial 16S rRNA was amplified using primers 515F (5′-GTGCCAGCMGCCGCGGTAA-3′) and 909R (5′-CCCCGYCAATTCMTTTRAGT-3′). The reaction conditions were as follows: 3 min of pre-denaturation at 94 °C; followed by 5 cycles of 30 s of denaturation at 94 °C, 20 s of annealing at 45 °C, 30 s of extension at 65 °C; followed by 20 cycles of 20 s of denaturation at 94 °C, 20 s of annealing at 55 °C, 30 s of extension at 72 °C; and final extension at 72 °C for 5 min, followed by cooling to 10 °C to end the reaction. Two rounds of amplification were performed. A 2% agarose gel electrophoresis was used to monitor the library size. Bright and clear bands meet the requirements for sequencing and are used for sequencing. After sequencing, OTUs were clustered based on 97% similarity, annotated and classified using the RDP database, and statistical and diversity analyses were conducted. For microbial community analysis, 500 mg of the compost sample was weighed and placed in a sterilized 2 mL centrifuge tube, followed by the addition of 1× PBS solution and shaking to mix evenly. Community structure analysis was carried out according to the above method. To predict the potential metabolic functions of the bacterial communities, PICRUSt2 was applied based on the OTU table, and functional annotation was performed against the KEGG database (version 94.2). The 16S rRNA sequencing was entrusted to Shanghai Sangon Biotech Co., Ltd. (Shanghai, China).

### 2.6. Data Analysis

The graphs and data analyses were performed using GraphPad Prism 9 software. The analysis of variance (ANOVA) was used to determine the significance of differences among the experimental samples (*p* < 0.01 was considered extremely significant; 0.01 < *p* < 0.05 significant, and *p* > 0.05 not significant).

## 3. Results

### 3.1. Growth Characteristics and Storage Conditions of NJ

**Figure 1:**
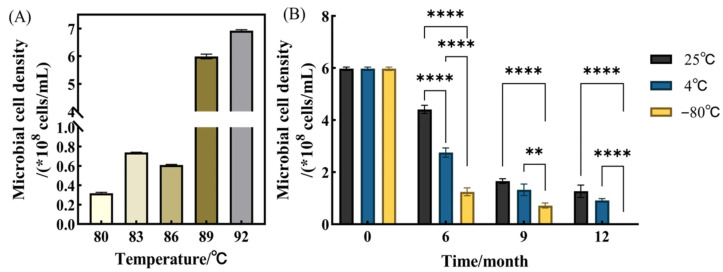
Microbial cell density: (**A**) Cultivation at different temperatures; (**B**) Storage at different temperatures (** indicates *p* < 0.01, and **** indicates *p* < 0.0001).

NJ was cultivated at different temperatures until microbial cell density had stabilized, with an optimal growth temperature range of 86–92 °C, achieving a maximum microbial cell density of 3.75 ± 0.28 × 10^8^ cells/mL (*p* < 0.05) (Figure 1A). The microbial community analysis was conducted on the consortium cultivated under the optimal temperature conditions (86–92 °C). Genus-level analysis identified 196 genera, predominantly hyperthermophilic, with the four most abundant being *Calditerricola* (46.3%), *Sphingomonas* (24.6%), *Paraburkholderia* (19.3%), and *unclassified* *Bacillales* (<2%). The post-storage regrowth capacity of NJ under different storage conditions was evaluated (Figure 1B). After storage at 25 °C for 6, 9, and 12 months, microbial cell density decreased to 73%, 27%, and 19% of the initial value, respectively, yet remained at the 10^8^ cells/mL level after 12 months. At 4 °C, microbial cell density declined to 44%, 20%, and 15% of the initial value over 6, 9, and 12 months, respectively, with the 12-month culture slightly below 108 cells/mL. Under −80 °C storage, microbial cell density decreased to 19% and 13% after 6 and 9 months, respectively, and no viable microorganisms were detected after 12 months. Statistical analysis indicated that both storage temperature and time significantly affected cell density (*p* < 0.001), with a significant temperature × time interaction; post hoc tests confirmed differences among temperatures at each time point (*p* < 0.05).

### 3.2. Effects of Inoculation at Different Stages with the NJ on Composting

**Figure 2:**
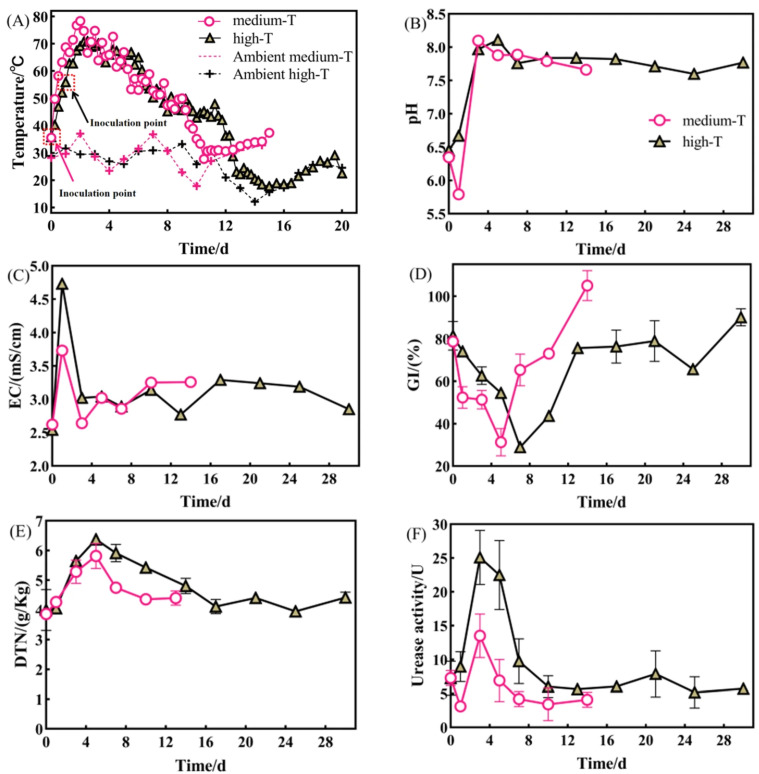
Physicochemical parameters of compost inoculated with NJ at different inoculation stages: (**A**) Temperature; (**B**) pH; (**C**) EC; (**D**) GI; (**E**) DTN; (**F**) Urease activity.

Medium-temperature stage inoculation of NJ significantly enhanced the composting heating rate and shortened composting time. The medium-T group rapidly increased to 78.3 °C at a rate of 36.96 °C/d, significantly higher than the peak of 70.9 °C observed in the high-T group (*p* < 0.05). Figure 2A presents the temperature variation during the first 20 days, as the compost temperature stabilized near ambient conditions thereafter (days 21–28). Composting was considered complete when the pile temperature stabilized near ambient conditions, along with stabilization of key physicochemical parameters, including EC, the C/N ratio, and GI [17]. Accordingly, the medium-T group completed composting in 14 days, over 50% faster than the high-T group (30 days). During the medium temperature stage, pH in the medium-T group transiently decreased from 7.5 to 6.9 within three days, whereas the high-T group maintained a weakly alkaline pH (7.4–8.5) (Figure 2B). EC trends were similar in both groups, rising rapidly at the medium-temperature stage, then gradually decreasing and stabilizing; the final EC was 3.26 mS/cm in the medium-T group vs. 2.85 mS/cm in the high-T group (*p* < 0.05) (Figure 2C).

Regarding the GI value, both groups exhibited a U-shaped trend. The minimum GI in the medium-T group was reached on day six, four days earlier than the lowest point observed in the high-T group. The final GI of the medium-T group reached 105%, significantly higher than the 90% in the high-T group (*p* < 0.05) (Figure 2D). While final DTN levels did not differ significantly, the medium-T group showed a delayed ammonium nitrogen peak and faster stabilization (Figure 2E). Urease activity in the medium-T group also displayed a lower peak and a more rapid decline compared with the high-T group (Figure 2F).

### 3.3. Verification of Medium-Temperature Stage NJ Inoculation Composting Effects

**Figure 3:**
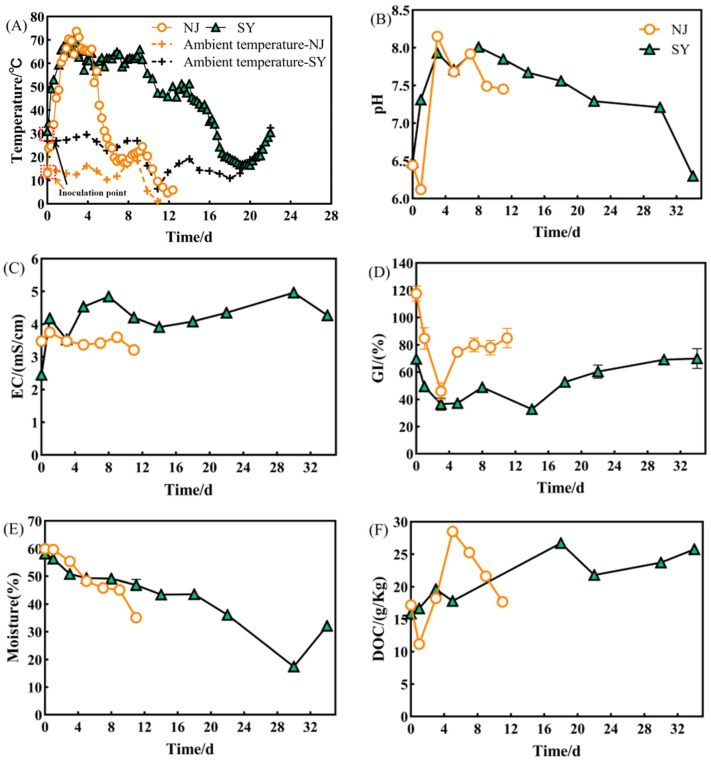

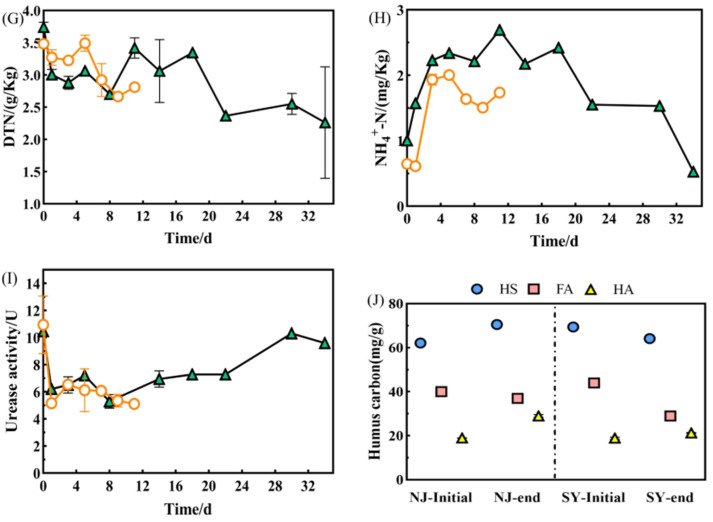
Physicochemical characteristics of compost inoculated with different microorganisms (NJ and SY) during composting. (**A**) Temperature; (**B**) pH; (**C**) EC; (**D**) GI; (**E**) Moisture content; (**F**) DOC; (**G**) DTN; (**H**) NH4^+^-N; (**I**) Urease activity; (**J**) Humus content.

Inoculation with NJ significantly accelerated the heating rate of composting and shortened composting time. On day one, the temperature rise rate of the NJ group (32.4°C/d) was 4 °C higher than that of the SY group, reaching a maximum of 73.7°C compared to 70.1°C in the SY group. Figure 3A presents the temperature variation during the first 28 days, as the compost temperature stabilized near ambient conditions thereafter (days 29–34). The ambient temperature curve is shown in Figure 3. A was used as the baseline for evaluating the temperature rise in the composting piles. Correspondingly, the NJ group exhibited a decrease in pH, indicating higher accumulation of organic acids during the initial composting stage (Figure 3B). Based on the stabilization of key indicators, the total composting time of the NJ group was only 11 days, which was 67% shorter than that of the SY group (34 days). Notably, the maturation period accounted for only 19% of the total composting time in the NJ group, compared to 41% in the SY group, which was the primary factor contributing to the shortened process.

Salinity and agricultural safety of compost products were significantly improved by the NJ inoculation. The EC values in the NJ group eventually stabilized at 3.34 mS/cm, significantly lower than the 4.27 mS/cm in the SY group (*p* < 0.001) (Figure 3C). Meanwhile, the GI in the NJ group increased at a faster rate, ultimately reaching 85%, while only 70% was achieved in the SY group, which was below the safety threshold (Figure 3D).

The reduction efficiency and process stability of composting were significantly enhanced by NJ inoculation. The overall reduction rate in the NJ group reached 48%, 7% higher than that of the SY group (41%), while dry matter reduction was 15%, exceeding that of the SY group by 7% (*p* < 0.05). Details are provided in the Supplementary Information (Table S4). In addition, the NJ group achieved a faster decrease in moisture content (Figure 3E).

The stable transformation of DOC was significantly promoted by NJ inoculation. The DOC content in the SY group continuously increased throughout the composting process, with the final value nearly 50% higher than that in the NJ group (*p* <0.001) (Figure 3F). Inoculation with NJ enhanced dissolved nitrogen retention, with DTN approximately 40% higher than that in the SY group (final content/initial content) (*p* >0.05) (Figure 3G). Specifically, the ammonium nitrogen accounted for 59% of DTN in the NJ group, whereas the prolonged high-temperature period in the SY group led to greater nitrogen loss through ammonia volatilization, resulting in a continuous decrease in ammonium nitrogen content (Figure 3H).

The rebound of urease activity in the late stage of composting was significantly inhibited by NJ inoculation. During the medium-temperature stage, both groups showed high urease activity, promoting urea hydrolysis and ammonium nitrogen accumulation (Figure 3I). After the high-temperature stage, urease activity in the NJ group was continuously suppressed, leading to the preservation of ammonium nitrogen, whereas a rebound of urease activity occurred in the SY group.

The accumulation and transformation of humus were significantly promoted by the NJ inoculation. By the end of composting, the humic substances (HS) content in the NJ group reached 114% of the initial value, achieving net accumulation, whereas only 93% was observed in the SY group (*p* < 0.001). Specifically, the humic Acid (HA) content increased by 56% in the NJ group, higher than that in the SY group (*p* < 0.001). In contrast, the fulvic acid (FA) decreased by 6% in the NJ group, much lower than the 31% observed in the SY group (*p* < 0.001) (Figure 3J), indicating a higher FA–to–HA conversion rate in NJ, which was the key pathway through which humus accumulation was realized.

### 3.4. Dynamics of Bacterial Community Structure During Composting

**Figure 4:**
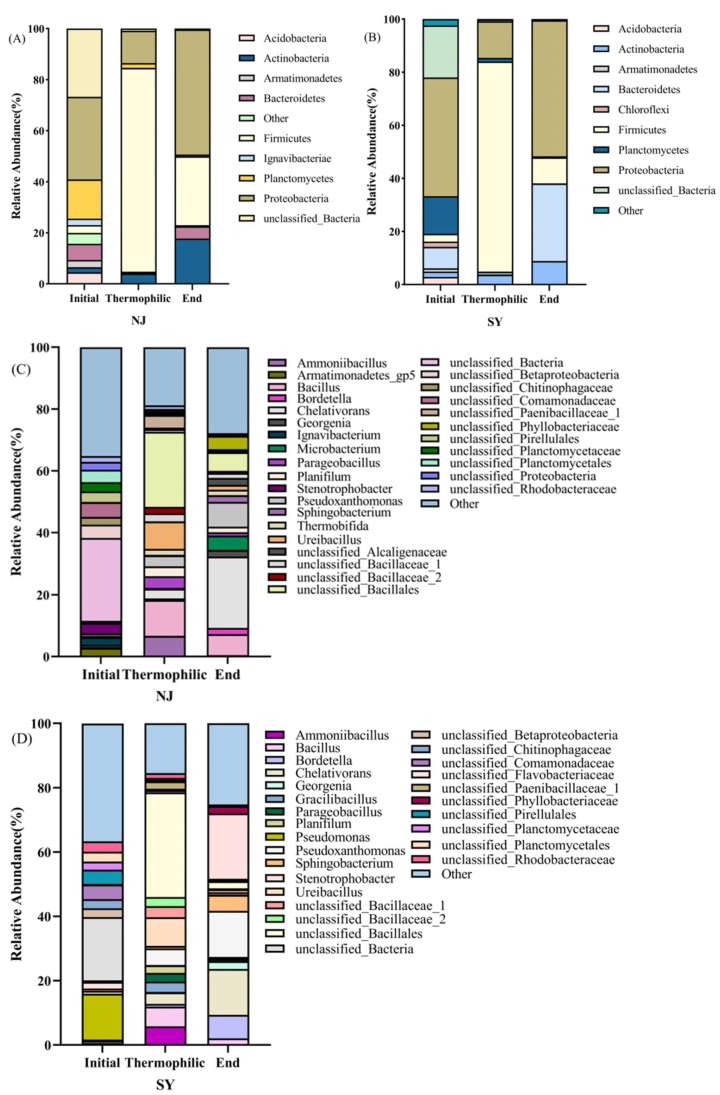
Microbial community structure at different taxonomic levels: phylum level, (**A**)—NJ; (**B**)—SY; genus level, (**C**)—NJ; (**D**)—SY. Microbial samples were collected on days 0, 4, and 11 for both groups.

The bacterial community structures of the NJ and SY groups (both inoculated at the initial stage) were compared at both the phylum and genus levels based on samples collected on days 0, 4, and 11 for both groups, representing the initial, thermophilic, and final stages of composting, respectively (Figure 4A–D). During the medium-temperature stage, both groups were dominated by *Proteobacteria* (NJ: 32.36%, SY: 44.68%) and *Planctomycetes* (NJ: 15.36%, SY: 14.2%). A higher proportion of *unclassified* phyla was observed in the NJ group (26.72%) than in the SY group (19.36%). During the thermophilic stage, *Firmicutes* rapidly became dominant in both groups (NJ: 79.87%, SY: 79.10%), accompanied by a marked decline in others. By the final stage, the succession of the communities diverged. In the NJ group, the abundance of *Firmicutes* decreased from 79.87% to 26.97%, while *Actinobacteria* markedly increased from 3.88% to 17.65%. The SY group exhibited a sharper reduction in *Firmicutes* abundance, from 79.10% to 9.76%, alongside the rebound of *Bacteroidetes* (from 1% to 29.8%) and *Proteobacteria* (from 13.79% to 51.22%). It should be noted that *Calditerricola* was identified under optimal cultivation conditions in Section 3.1, whereas it was not detected in the composting samples shown in Figure 4. This difference may be attributed to the low inoculation proportion (0.31%, *v*/*v*) and the complexity of the indigenous microbial community in the compost matrix.

At the genus level, the microbial community composition in both groups was similar during the initial stage, dominated by *unclassified_Bacteria*, while *Pseudomonas* showed a higher relative abundance in the SY group (14.26%). During the thermophilic stage, both communities were restructured, with the proportions of the original dominant genera decreasing, while thermotolerant genera such as *unclassified_Bacillales*, *Ureibacillus*, and *Bacillus* were significantly enriched. The abundance of *Ammoniibacillus* increased markedly in the SY group (from 0.07% to 5.8%). By the final stage, *Chelativorans*, *Pseudoxanthomonas*, and *unclassified* genera became dominant in both groups, whereas the *unclassified_Flavobacteriaceae* accounted for 20.34% and was exclusively detected in the SY group.

## 4. Discussion

### 4.1. Higher Regrowth Capacity of NJ Stored at 25 and 4 °C than −80 °C

The long post-storage regrowth capacity of NJ was evaluated at −80, 4, and 25 °C. NJ maintained a microbial cell density of approximately 10^8^ cells/mL after nine months of storage at 25 or 4 °C, whereas storage at −80 °C resulted in a decrease below 10^8^ cells/mL within six months and no detectable viable cells after 12 months. This observation is consistent with the findings of Unsworth et al. [18] that ambient temperatures for hyperthermophiles may have effects similar to refrigerated temperatures for mesophilic microorganisms. Similarly, Oshima et al. [12] reported that the hyperthermophilic genus *Calditerricola* could not be preserved for the long term at −80 °C, suggesting that certain hyperthermophiles may be susceptible to cryopreservation-induced stress. Flores et al. [19] further emphasized that both the cultivation and preservation of hyperthermophiles remain challenging, with no universally optimal methods due to species-specific physiological characteristics.

In addition, the suitability of conventional cryopreservation strategies for hyperthermophiles remains uncertain. Although −80 °C storage with cryoprotectants (e.g., glycerol) is widely used for many microorganisms, previous studies have shown that such methods may not be universally applicable to hyperthermophiles and may even lead to loss of viability in certain species [12]. Short-term handling of hyperthermophilic cultures at moderate temperatures has been reported to be feasible, whereas the effects of long-term storage under such conditions remain less well-understood [20]. Therefore, in the present study, a consistent preservation procedure was applied across all temperature treatments.

The reduced regrowth capacity observed at ultra-low temperatures may be associated with cryo-induced cellular stress. Freezing can cause membrane phase transitions, protein destabilization, and intracellular ice crystal formation, which negatively affect cellular integrity and subsequent recovery [21]. Although hyperthermophiles possess membrane lipids and protein structures adapted to high temperatures, these features may also increase their sensitivity to freezing-related stress [6]. Therefore, the limited suitability of −80 °C storage for NJ under the applied conditions may be related to these physiological characteristics, although direct viability assays were not conducted in this study. Temperature shifts may also impose selective pressures on thermophilic microorganisms, potentially influencing their physiological stability. From an application perspective, the regrowth performance of NJ after storage should be considered in relation to its functional recovery under dynamic composting conditions, where different temperature stages may affect microbial adaptation and activity.

### 4.2. Medium-Temperature Stage Inoculation of NJ Is More Effective for Composting

The comparison of the inoculation stages revealed that NJ inoculation at the medium-temperature stage was more effective than inoculation at the high-temperature stage (>55°C). This advantage could be attributed to the ecological and physiological contexts of the medium-temperature stage. At this stage, ecological competition is relatively weak, and nutrients are abundant, providing a favorable window for exogenous consortium establishment. During microbial adaptation, active proliferation may facilitate rapid colonization of NJ populations. Medium-temperature stage inoculation allowed the functional consortium to establish dominance before ecological niches became saturated, thereby activating key degradation pathways during the mesophilic phase.

The comparison of the inoculation stages showed that inoculation at the medium-temperature stage resulted in better composting performance than inoculation at the high-temperature stage (>55 °C). This advantage can be interpreted from a process-based ecological perspective involving establishment timing, substrate availability, and environmental stress. During the medium-temperature stage, relatively favorable environmental conditions, including moderate temperature, sufficient moisture, and higher availability of readily degradable substrates, may facilitate the establishment and activity of the introduced microbial consortium. Previous studies have also suggested that microbial colonization and proliferation are more efficient under conditions with lower environmental stress and greater substrate accessibility, which may enhance the effectiveness of inoculation [22].

In contrast, inoculation during the high-temperature stage may face multiple constraints. At this stage, easily degradable substrates are largely consumed, leaving more recalcitrant fractions such as cellulose and lignin, which are more difficult to utilize. In addition, elevated temperatures and reduced moisture may impose physiological stress on the introduced microorganisms, potentially delaying their adaptation and functional expression. Such temperature-induced stress responses and shifts in metabolic activity have been widely reported in thermophilic systems [23,24,25]. The nitrogen dynamics further support this interpretation. In the medium-temperature group, transient pH decreases may have suppressed early ammonia volatilization, while improved nitrogen retention was observed. In addition, differences in the inoculation stage influenced the heating rate and thermophilic temperature, which in turn could affect urease activity. A lower peak and faster decline of urease activity may partly explain the differences in nitrogen transformation between treatments [26]. These effects may be related to more balanced process conditions and more efficient coupling of carbon and nitrogen transformations during this stage.

Overall, the superiority of medium-temperature inoculation observed in this study is likely a result of multiple interacting factors, including environmental conditions, substrate availability, and microbial response dynamics.

### 4.3. NJ Significantly Enhanced Composting Efficiency and Product Quality

The NJ consortium reshaped the composting track by rapidly raising the temperature and driving more thorough organic matter decomposition. The transient decrease in pH observed at the medium-temperature stage indicates that part of the nitrogen retention in the NJ group was attributable to the suppression of ammonia volatilization through acidic conditions during the medium-temperature stage [27]. In the later phase, nitrogen retention could be attributed to reduced urease activity, which suppressed urea hydrolysis and limited nitrogen losses [28].

The lower EC value observed in the NJ compost not only indicates a safer product quality but also reflects differences in composting processes between the groups. EC is primarily influenced by the accumulation of soluble salts, including inorganic ions and low-molecular-weight organic compounds. The lower EC in the NJ group may be attributed to more efficient organic matter mineralization and nutrient transformation in the hyperthermophile-inoculated composting process [29], which together reduce the accumulation of soluble intermediates and inorganic nitrogen species, particularly ammonium, during composting [30]. Consequently, the final EC values met the commonly accepted non-phytotoxic threshold (EC < 4.0 mS/cm) [31].

In addition to salinity- and nutrient-related indicators, heavy metal behavior represents an important aspect of compost quality. Apparent increases in heavy metal concentrations are commonly observed during composting due to water loss, organic matter mineralization, and volume reduction [32]. However, such concentration effects do not necessarily indicate increased environmental risk, as mobility and bioavailability are more relevant to compost safety. Composting is widely recognized as an effective approach for heavy metal stabilization through enhanced humification, whereby humic and humus-like substances immobilize metal ions [33,34]. In this study, the improved organic matter degradation and HA observed in the NJ group compost may be conducive to heavy metal stabilization. Moreover, the establishment of hyperthermophiles could further promote metal association with organic matrices via enhanced humification and extracellular polymeric substance production [10,35]. Accordingly, the NJ group is unlikely to adversely affect compost safety with respect to heavy metals, although direct measurements of metal speciation and bioavailability are required to substantiate these potential effects.

At the microbial level, the composting performance of the NJ group is closely associated with community dynamics and functional gene expression. During the medium-temperature stage, NJ was dominated by *Proteobacteria* (32.36%) and *Planctomycetes* (15.36%), with a relatively high proportion of *unclassified* phyla (26.72%), indicating early-stage community establishment. In the thermophilic stage, *Firmicutes* rapidly became dominant (79.87%), accompanied by enrichment of thermotolerant genera such as *Bacillus*, *Ureibacillus*, and *unclassified_Bacillales*. These genera are capable of secreting extracellular enzymes and forming heat-resistant spores, contributing to accelerated organic matter degradation, pathogen suppression, and humus accumulation [36,37]. In the cooling and maturation stages, the NJ group developed distinct dominant taxa. *Firmicutes* decreased from 79.87% to 26.97%, *Actinobacteria* increased from 3.88% to 17.65%, and dominant genera included *Chelativorans* and *Pseudoxanthomonas*, both associated with cellulose degradation and denitrification [38,39].

### 4.4. Temperature-Mediated Functional Differentiation Between Hyperthermophilic and Thermophilic Composting

In conventional thermophilic composting systems (cTC), microbial community succession and functional processes have been thoroughly studied. Composting temperatures in cTC systems typically reach and stabilize at around 50–65 °C [3], under which thermophilic bacteria such as *Bacillus*, *Ureibacillus*, and other members of *Firmicutes* dominate [40]. These microorganisms are highly effective in utilizing easily degradable organic matter, including carbohydrates and proteins, and play a central role in driving organic matter mineralization during the thermophilic phase [36]. Meanwhile, nitrogen cycling processes such as ammonification, nitrification, and denitrification remain partially active, which may lead to nitrogen loss through ammonia volatilization and gaseous emissions [41]. In addition, microorganisms capable of degrading more recalcitrant substrates, such as complex lignocellulosic fractions and microbial necromass, show limited metabolic activity. Consequently, humification largely depends on prolonged composting time and secondary transformations during maturation.

Notably, the advantage of ultra-high-temperature composting (UHTC) compared with cTC systems lies in two key aspects. First, specialized microorganisms capable of degrading recalcitrant organic matter are present, enabling efficient breakdown of complex substrates [42]. In this study, NJ-guided composting increased the abundance of *Actinobacteria*. These microorganisms facilitated the decomposition of recalcitrant fractions, stabilized the compost, and enhanced nitrogen retention by breaking down complex substrates into small-molecule organic acids, which reduced ammonia volatilization and supported nitrification [43]. Second, certain hyperthermophilic and thermotolerant populations can survive and remain metabolically active under extreme thermal stress, sustaining high-temperature conditions. Representative taxa include thermotolerant *Firmicutes*, such as *Bacillus*, *Virgibacillus*, and *Calditerricola*, which possess thermostable enzymatic systems adapted to prolonged high temperatures [42]. Together, these two mechanisms allow UHTC to achieve enhanced organic matter mineralization and sustain high-temperature conditions compared with cTC systems.

Under UHTC conditions, the accelerated transformation of organic matter provides favorable conditions for humic substance formation. As a result, humification-related processes occur earlier and more intensively than in cTC systems, as reflected by increased HA content and humification indices [44]. At the same time, the suppression of temperature-sensitive nitrification and denitrification pathways reduces nitrogen loss via gaseous emissions, contributing to improved nitrogen retention [45]. Moreover, sustained ultra-high temperatures enhance compost pathogen reduction, promoting the removal of antibiotic residues and the reduction of antibiotic resistance genes that are often insufficiently eliminated under cTC systems [46].

Together, these mechanisms not only drive enhanced organic matter mineralization and maintain high temperatures but also explain why inoculation with hyperthermophiles at the initial stage accelerates functional activity, improving composting efficiency and product quality.

## 5. Conclusions

In this study, NJ maintained higher post-storage regrowth capacity after six months at 25 and 4 °C than at −80 °C. Inoculation at the medium-temperature stage proved more effective than inoculation at the high-temperature stage, resulting in faster temperature increase, shorter composting time, improved nitrogen retention, and enhanced humification. Compared with the commercial inoculant, NJ further improved compost maturity and overall process performance. These effects were associated with shifts in microbial community composition, particularly the enrichment of thermophilic taxa. Overall, this study highlights that both post-storage regrowth capacity and the inoculation stage are key factors influencing the functional performance of hyperthermophilic inoculants in sludge composting. However, the functional gene expression patterns and the mechanism underlying microbial activity during storage and inoculation processes remain insufficiently resolved and require further investigation.

## Data Availability

The original contributions presented in this study are included in the article/Supplementary Materials. Further inquiries can be directed to the corresponding author.

## Supplementary Materials

The following supporting information can be downloaded at: https://www.mdpi.com/article/10.3390/microorganisms14051064/s1, Text S1: Detailed method for the determination of total humic substances; Figure S1: Composting device; Table S1: Working condition parameters; Table S2: Composition and parameters of pilot-scale composting piles inoculated with NJ at different temperatures; Table S3: Pile composition and parameters of pilot-scale composting with NJ and SY inoculants; Table S4: Reduction indicators of pilot-scale composting.
