# Supplementary material for: Temperature-Driven Responses and Contributions of Hyperthermophiles: Linking Storage and Inoculation Strategies in Municipal Sludge Composting
Source: Microorganisms. 2026 May 8;14(5):1064. doi: 10.3390/microorganisms14051064 (PMC13209927; doi:10.3390/microorganisms14051064)
Supplement: Supplementary file 1 [file microorganisms-14-01064-s001.zip › microorganisms-4297011-supplementary.pdf]

Supplementary Material

Text S1: The method for determining total humus is as follows-Take 2.00 g of the dried and ground sample (filtered through a 40-mesh sieve), add 20 ml of extraction solution (0.1 mol/L NaOH + 0.1 mol/L $Na_2P_2O_7$; V/V = 1:1), shake at 250 rpm/min for 2 hours, centrifuge at 4000 rpm/min for 10 minutes, and collect the supernatant. The filter residue is treated in the same way three times, and the filtrate is combined to obtain total humus. The determination methods for humic acid and fulvic acid are as follows: Acidify the filtrate with 6 mol/L hydrochloric acid to a pH of 1-2, shake thoroughly, and let it stand at room temperature overnight. Centrifuge at 4000 rpm/min for 10 minutes, and the upper clear liquid is fulvic acid (FA), while the lower precipitate is humic acid (HA). The humic acid precipitate is dissolved with 0.1 mol/L KOH and measured, and the humus content is characterized by separately determining the TOC content of HS, FA, and HA.

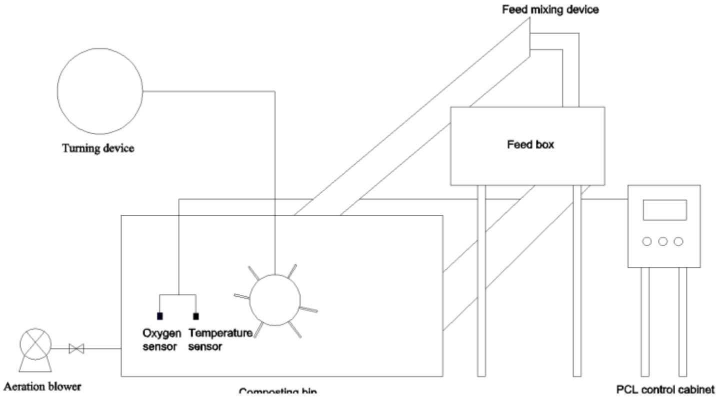

Figure S1. Composting device

Table S1. Working condition parameters

Parameters	Values
Turning frequency (times/day)	2
Single turning time (min)	20
Aeration flow rate (L/min/kg)	0.65
Oxygen concentration control range(%)	6-14

Table S2. Composition and parameters of pilot-scale composting piles inoculated with NJ at different temperatures

Group	medium-T(mean ± SD, n=3)	high-T(mean ± SD, n=3)
Total mass (kg)	320 ± 0.2	320 ± 0.2
Sludge (kg)	175 ± 0.3	175 ± 0.3
Straw (kg)	115 ± 0.0	115 ± 0.0
Pure water (L)	29 ± 0.1	29 ± 0.1
Bacterium (L)	1 ± 0.1(NJ，0.31%)	1 ± 0.1(NJ，0.31%)
Concentration of bacterium (cells/ml)	$1.6*10^8 \pm 0.0$	$1.6*10^8 \pm 0.0$
total amount of added bacterial（cells）	$1.6*10^{11} \pm 0.0$	$1.6*10^{11} \pm 0.0$
Inoculation stage	Initial	The temperature of the pile > 55℃

Table S3. Pile composition and parameters of pilot-scale composting with NJ and SY inoculants.

Group	NJ(mean ± SD, n=3)	SY(mean ± SD, n=3)
Total mass (kg)	320 ± 0.2	320 ± 0.2
Sludge (kg)	175 ± 0.3	175 ± 0.3
Straw (kg)	115 ± 0.0	115 ± 0.0
Pure water (L)	29 ± 0.1	29.7 ± 0.0
Bacterium (L)	1 ± 0.1(NJ, 0.31%)	0.32 ± 0.1(SY, 0.1%)
Concentration of bacterium (cells/ml)	$1.6*10^{8}$ ± 0.0	$5*10^{8}$ ± 0.0
Total amount of added bacterial（cells）	$1.6*10^{11}$ ± 0.0	$1.6*10^{11}$ ± 0.0

Table S4. Reduction indicators of pilot-scale composting.

Parameter	NJ(mean ± SD, n=3)	SY(mean ± SD, n=3)
Initial mass (kg)	320.0 ± 0.2 a	320.0 ± 0.2 a
Final mass (kg)	167.5 ± 0.4 a	191.1 ± 0.4 b
Overall reduction rate (%)	48	41
Initial moisture content (%)	60	58
Final moisture content (%)	35	35
Mass reduction rate (%)	15	9

Different letters in the same column indicate significant differences between groups ($p < 0.05$, t-test).

Values are presented as mean ± SD (n = 3).
